# A comparative study of virus nucleic acid re-positive and non-re-positive patients infected with SARS-CoV-2 Delta variant strain in the Ningxia Hui Autonomous Region

**DOI:** 10.3389/fpubh.2022.1023797

**Published:** 2022-12-13

**Authors:** Jing Wang, Shu-Xiang Zhang, Jian-Rong Na, Li-Ling Zhang, Yin-Hao Zhang, Jiao-Jiao Chu, Lei Guo, Mei Yan, Yu-Ting Li, Wei Zhou

**Affiliations:** ^1^Clinical Medical College, Ningxia Medical University, Yinchuan, China; ^2^Department of Respiratory and Critical Care Medicine, Ningxia Medical University General Hospital, Yinchuan, China; ^3^Department of Medical Education, Fourth People's Hospital of the Ningxia Hui Autonomous Region, Yinchuan, China; ^4^Ningxia Center for Disease Control, Yinchuan, China; ^5^Department of Comprehensive Internal Medicine, Fourth People's Hospital of the Ningxia Hui Autonomous Region, Yinchuan, China

**Keywords:** Delta variant infection, virus nucleic acid re-positive, antibody level, virus nucleic acid CT value, COVID-19 vaccine

## Abstract

**Objective:**

This study aimed to provide a basis for epidemic prevention and control measures as well as the management of re-positive personnel by analyzing and summarizing the characteristics of re-positive patients with severe acute respiratory syndrome coronavirus 2 (SARS-CoV-2) Delta variant infections discharged from a hospital in the Ningxia Hui Autonomous Region in 2021.

**Methods:**

This case-control study included a total of 45 patients with Delta variant infections diagnosed in the Fourth People's Hospital of the Ningxia Hui Autonomous Region between October 17 and November 28, 2021. Based on the nucleic acid test results post-discharge, the patients were dichotomized into re-positive and non-re-positive groups. Based on the time of the first re-positive test, the re-positive group was further divided into <7 and ≥7 days groups to compare their clinical characteristics and explore the possible influencing factors of this re-positivity.

**Results:**

Of the 45 total patients, 16 were re-positive (re-positivity rate: 35.6%), including four patients who were re-positive after 2 weeks (re-positivity rate: 8.8%). The median time of the first re-positive after discharge was 7 days (IQR: 14-3). The re-positive group was younger than the non-re-positive group (35 vs. 53, *P* < 0.05), had a higher proportion of patients who were not receiving antiviral therapy (56.2 vs. 17.2%, *P* < 0.05). The median CT value of nucleic acid in the re-positive group was considerably greater than that at admission (36.7 vs. 22.6 *P* < 0.05). The findings demonstrated that neutralizing antibody treatment significantly raised the average IgG antibody level in patients, particularly in those who had not received COVID-19 vaccine (*P* < 0.05). The median lowest nucleic acid CT value of the ≥7 days group during the re-positive period and the immunoglobulin G (IgG) antibody level at discharge were lower than those in the <7 days group (*P* < 0.05). When compared to the non-positive group, patients in the ≥7 days group had a higher median virus nucleic acid CT value (27.1 vs. 19.2, *P* < 0.05) and absolute number of lymphocytes at admission (1,360 vs. 952, *P* < 0.05), and a lower IgG antibody level at discharge (*P* < 0.05).

**Conclusions:**

In conclusion, this study found that: (1) The re-positivity rate of SARS-CoV-2 Delta variant infection in this group was 35.6%, while the re-positivity rate was the same as that of the original strain 2 weeks after discharge (8.0%). (2) Young people, patients who did not use antiviral therapy or had low IgG antibody levels at discharge were more likely to have re-positive. And the CT value of nucleic acid at the time of initial infection was higher in re-positive group. We speculated that the higher the CT value of nucleic acid at the time of initial infection, the longer the intermittent shedding time of the virus. (3) Re-positive patients were asymptomatic. The median CT value of nucleic acid was > 35 at the re-positive time, and the close contacts were not detected as positive. The overall transmission risk of re-positive patients is low.

## Introduction

Coronavirus disease of 2019 (COVID-19) is an acute respiratory infectious disease caused by severe acute respiratory syndrome coronavirus 2 (SARS-CoV-2) (1). Owing to the transmissibility and adaptability of SARS-CoV-2, it spread rapidly worldwide and underwent mutations, transforming into several dominant pedigrees within 2 years (2). The main variants include B.1.1.7 (Alpha), B.1.351 (Beta), P.1 (Gamma), and B1.617.2 (Delta), which have become major sources of infections in more than 90 countries since May 2021 (3). The Delta variant was first discovered in India in October 2020 and showed quick transmission, strong pathogenicity, and rapid disease progression, which resulted in a new round of the global epidemic (4). This variant also caused a large-scale epidemic in China in 2021. After the SARS-CoV-2 infected persons were cured, cases of virus nucleic acid test positivity (re-positive) after discharge were reported occasionally. These cases have increased the complexity of epidemic control and aroused widespread concern.

A study in Brunei (5) reported that the original strain causing COVID-19 had a re-positivity rate of ~19.8% (21/106). In that study, age was the only significant risk variable, with people ≥60 years showing the highest risk of re-positivity. A study from Guangzhou, China, reported (6) a re-positivity rate of the original strain infection of ~21.2% (157/745), suggesting that young patients without severe clinical symptoms had a high risk of re-positivity; however, their family members and close contacts showed negative nucleic acid test results. Therefore, the risk of transmission was extremely low. However, a recent study (7) on the Delta variant infected individuals showed a re-positivity rate of ~61.4% (514/837), which was significantly higher than that of the original strain, indicating that the combination of unvaccinated status, older age, and underlying disease could be a prognostic indicator in distinguishing patients with potentially high concentrations of viral RNA. Since the Delta variant has higher infectivity and viral load than the original strain (4), it may have a longer viral shedding time, leading to a significantly higher re-positive rate. These studies have found that the re-positivity proportion of recovered COVID-19 patients was higher after discharge, and the re-positivity rate of SARS-CoV-2 Delta variant infection increased significantly. However, to date, most of the existing studies are about the re-positivity of the original strain, and there are still few reports on the re-positive of the SARS-CoV-2 Delta variant strain.

From October 17, 2021, to November 28, 2021, a total of 45 patients with SARS-CoV-2 Delta variant strain infection were diagnosed in Ningxia Hui Autonomous Region, China, and all of them were discharged from the hospital. To understand the re-positivity rate and transmission risk of Delta variant strain infection in Ningxia Hui Autonomous Region after discharge, this study retrospectively analyzed all patients, compared the clinical and epidemiological data of re-positive and non-re-positive patients, explored the influencing factors of re-positive and speculated the time standard of true re-positive. Provide a basis for epidemic prevention and control and management measures for re-positive personnel.

## Materials and methods

### Study design and sample size

**Figure 1:**
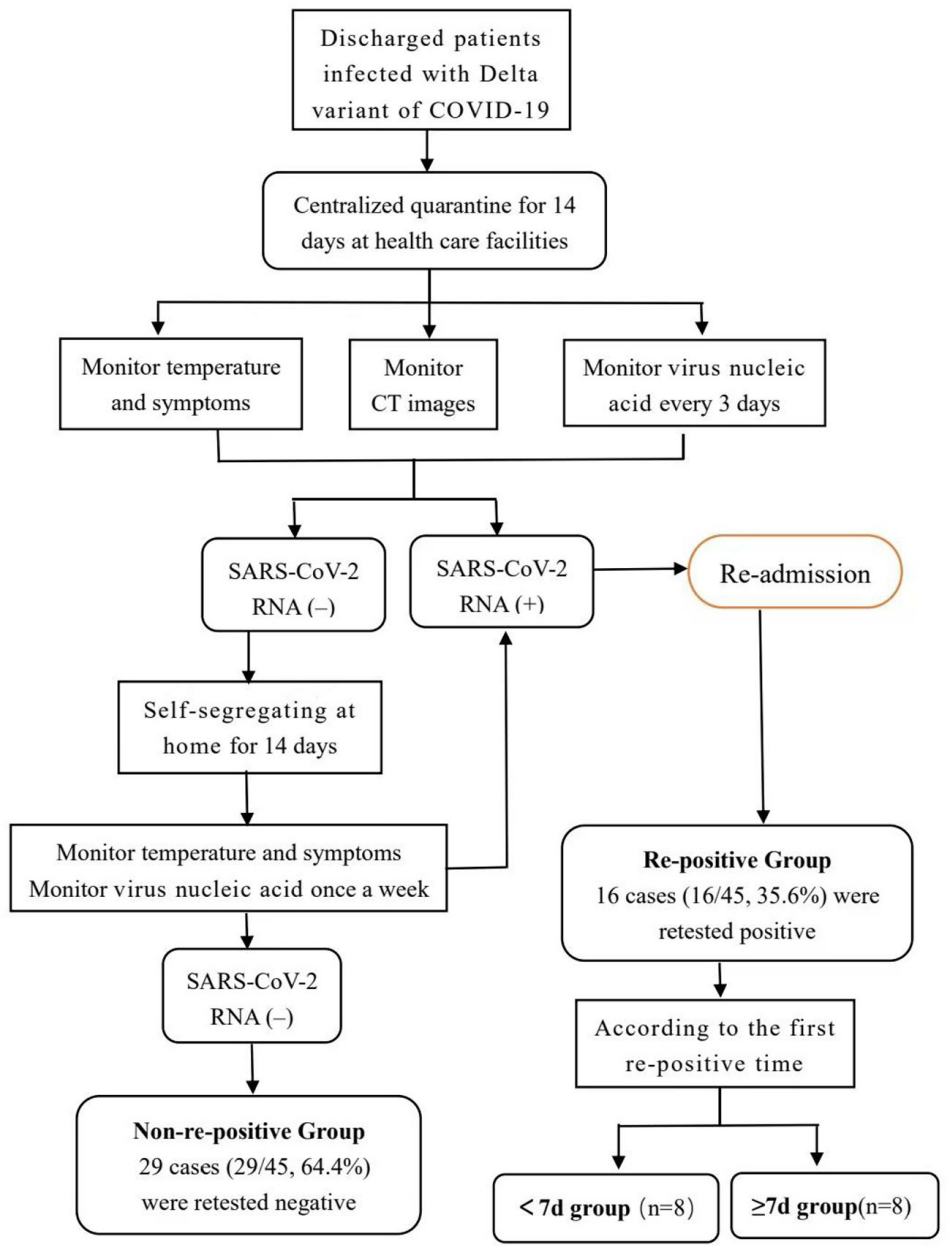
Follow up flow chart of 45 patients with delta variant of COVID-19 diagnosed and discharged from hospital in Ningxia Hui Autonomous Region. As shown in the figure, all discharged patients are quarantined in health care facilities for 14 days. Virus nucleic acid monitor every 3 days during centralized isolation. The patients with positive virus nucleic acid tests were re-admitted to hospital for further isolation and observation. Patients with negative virus nucleic acid tests were quarantined at home for the next 14 days. Based on the results of the nucleic acid detection after discharge, the patients were divided into two groups: re-positive and non-re-positive. Based on the time taken to the first re-positive test, the re-positive group was further divided into the <7 and ≥7 days groups. Patients in the re-positive group were re-admitted to the designated hospital for further observation, and they were discharged again when they met the discharge criteria. Patients who had finished the processes above were included in our study.

This retrospective case-control study included a total of 45 patients diagnosed with a Delta mutant infection and hospitalized in isolation at the Fourth People's Hospital of the Ningxia Hui Autonomous Region between October 17 and November 5, 2021, and who met the discharge criteria of the “Novel Coronavirus Pneumonia Treatment Protocol (trial vision 8).” The discharge criteria were as follows (8): maintaining normal body temperature for >3 days, significantly improved respiratory symptoms, pulmonary imaging showing significant improvement of acute exudative lesions, and two consecutive negative nucleic acid tests of respiratory tract samples (with at least 24 h between sampling times). Delta variant infection was confirmed by viral genetic testing by the National Center for Disease Control and the Ningxia Hui Autonomous Region Center for Disease Control. All patients had a clear source and chain of transmission. In accordance with the requirements of the guidelines, all discharged patients were required to undergo 14 days of quarantine in designated healthcare facilities (8). During the isolation observation period, the recovered patients were tested for nucleic acids every 3 days. Self-segregation at home for 14 days after the centralized quarantine was lifted. Continue to observe the clinical symptoms and virus nucleic acid test results. Re-positive patients were re-admitted to hospital for isolation observation and treatment, and their close contacts were followed up. Figure 1 shows the flowchart of the management of discharged patients. See the process in Figure 1.

Based on the results of the nucleic acid detection after discharge, the patients were divided into two groups: re-positive and non-re-positive. Based on the time taken to the first re-positive test, the re-positive group was further divided into the <7 and ≥7 days groups. General and clinical data were collected, including patient's sex, age, underlying diseases, duration of hospital stay, time to first nucleic acid negative conversion, therapeutic drugs, antibody positivity rate, antibody levels at admission, discharge, and 2 weeks after discharge, nucleic acid CT value, and T lymphocyte subsets at admission.

### Definitions

Re-positivity diagnostic criteria of SARS-CoV-2 nucleic acid test: Among COVID-19 patients who were discharged after meeting discharge criteria, the cycle threshold (CT) value of real-time reverse transcriptase PCR (RT-PCR) detection on the nasal or pharyngeal swab < 40 was defined as re-positive (8–10). The time of the first re-positivity was defined as the number of days between the sampling date of the first re-positive nucleic acid test after discharge and the date of discharge from the hospital after meeting the discharge criteria.

Diagnosis and classification of COVID-19 as following (8). (1) Mild cases who have mild clinical symptoms but no pneumonia manifestation in imaging. (2) Moderate cases who have fever and mild respiratory symptoms (cough, sore throat, respiratory tract symptoms, etc), multiple patchy shadowing and ground-glass opacity in lung computed tomography (CT), and normal range of vital signs. (3) Severe cases in adults who exhibit any of the symptoms listed below: respiratory distress (respiratory rate ≥ 30 breaths/min), and/or SpO_2_ ≤ 93%, and/or PaO_2_/FiO_2_ ≤ 300 mmHg (1 mmHg = 0.133 kPa), and/or pulmonary lesions increased >50% within 24–48 h in imaging. Severe cases in children who exhibit any of the symptoms listed below: high fever persisted for more than 3 days, respiratory distress (< 2 months old, RR ≥ 60 breaths/min; 2–12 months old, RR ≥ 50 breaths/min; 1–5 years old, RR ≥ 40 breaths/min; >5 years old, RR ≥ 30 breaths/min. SaO_2_ ≤ 93% under resting condition) and need to assist breathing (nasal alar flapping, triple concave sign). In addition, have symptoms and signs such as drowsiness, convulsions, anti-feedant or feeding difficulties, and signs of dehydration. (4) Critical cases who exhibit any of the symptoms listed below: respiratory failure need mechanical ventilation, and/or shock, and/or combined other organ failure requiring intensive care unit.

### Novel coronavirus nucleic acid detection method

The nasal and pharyngeal swabs of 45 patients were collected by professionals during the period of diagnosis and isolation observation. The tests were performed using the reagents approved by the state (extraction reagent: Jiangsu shuoshi reagent; amplification reagent: Shanghai Zhijiang reagent). Total RNA was extracted within 2 h and two target genes [the open reading frame 1ab (ORF1ab) and nucleocapsid protein (N)], were amplified and tested by real-time reverse transcriptase PCR (RT-PCR) polymerase chain reaction. The amplification conditions were reverse transcription at 42°C for 5 min, pre- denaturation at 95°C for 10 s, followed by 40 cycles of denaturation at 95°C for 10 s, expansion at 60°C, and collection of fluorescent signals for 45 s. Nucleic acid CT values < 40 were defined as positive.

### Detection of IgG and IgM antibodies to the novel coronavirus

A novel coronavirus antibody detection kit (colloidal gold method) [Inot (Tangshan) Biotechnology Co., Ltd. National Machinery label 20203400177] was used to detect novel coronavirus-specific IgM and IgG antibodies from peripheral blood samples. The method was as follows: 10 μL of serum was added to the sample hole of the test card; 80 μL of sample diluent was then added to the diluent on the test card, and the results were observed after 15 min. The kit instructions were strictly followed. The interpretation of the results is described as follows: S/CO values >1 and < 1 were considered positive (+) and negative (-), respectively.

### T lymphocyte subsets detection method

Within 24 h after admission, fasting venous blood was taken to detect T lymphocyte subsets. AgilentNovoCyte flow cytometry (USA) and matching T lymphocyte detection kit (flow cytometry-FITC/PE/PerCP/APC) were used. At room temperature, the antibody reagent in the 20 uLT lymphocyte detection kit was applied to the bottom of the labeled flow tube. Add 50 uL well-mixed anticoagulant human peripheral blood to the bottom of the test tube, cover, shake gently on the whirlpool mixer for 5 s, incubate for 15 min at room temperature (18–25°C), add 450 uL hemolysin, cover, gently shake on the whirlpool mixer for 5 s, incubate 15 min at room temperature (18–25°C), and then obtain and analyze it on flow cytometry. Analysis was carried out with supporting NovoExpress software.

### Statistical analysis

IBM SPSS Statistics for Windows, version 26.0 was used to perform the statistical analyses. Continuous variables were expressed as median and interquartile range (IQR) values and compared by Mann-Whitney *U*-test. Counting data were expressed as number (%) and compared by χ^2^ or Fisher's exact probability tests. Statistical tests were two-sided, and the significant level (α) in our study is 0.01 The *p*-values of < 0.05 were considered to indicate statistical significance.

## Results

### General characteristics

This study included 45 COVID-19 patients who were infected with the Delta variant. Of these, 17 cases were men (37.8%) and 28 cases were women (62.2%). The ages ranged from 33 days to 80 years, with an average age of 44 years. One case had mild symptoms, 43 cases had moderate symptoms (95.6%), and one case had severe symptoms. One case received one dose of the COVID-19 vaccine, 35 cases received two doses, and one case received three doses. While the other eight were not. Among the 45 cases, 16 were re-positive, corresponding to a re-positivity rate of 35.6%. Four cases were re-positive after 2 weeks (re-positivity rate 8.8%). The non-re-positive group included 29 cases (64.4% of the total cases). The time range of the first re-positive after discharge was 3–30 days, with an median time of 7 days (IQR: 14–3). The first re-positivity occurred in <7 days in eight cases (50%) and ≥7 days in eight cases (50%), including two cases that remained re-positive for >2 months. Of these, one case became negative after 65 days, while the other case became negative after 74 days. Among the samples tested, the re-positivity rate of nasal swabs was significantly higher than that of pharyngeal swabs. Nasal and throat swab nucleic acid tests in three patients (18.8%) simultaneously re-positive. Pharyngeal swabs were re-positive and the nasal swabs from the same patient were negative in two cases (12.5%). Nasal swabs were re-positive while the pharyngeal swabs from the same patient were negative in 11 cases (68.8%). While 16 re-positive cases were re-admitted to the hospital for isolation and observation, none showed clinical symptoms and were not administered any special treatment.

### Clinical characteristics of the re-positive and non-re-positive groups

The median age of the re-positive group was significantly lower than that of the non-re-positive group (35 vs. 53, *P* < 0.05). The proportion of patients in the non-re-positive group who were administered antiviral treatment was significantly higher than those in the re-positive group (*P* < 0.05; Table 1).

**Table 1:** Comparisons of clinical characteristics between the re-positive and non-re-positive groups^a^.

**Variables**	**Re-positive group (*n* = 16)**	**Non-re-positive group (*n* = 29)**	***P*-value**
Age (years)	35 (56–13.5)	53 (59–40)	0.049
Gender [Male (%)]	6 (35.3)	11 (64.7)	0.98
Length of hospitalization (days)	17.5 (22.0–12.8)	19.0 (21.0, 17.0)	0.505
Fever [cases (%)]	11 (68.8)	23 (79.3)	0.483
With underlying diseases [cases (%)]	5 (31.3)	11 (37.9)	0.654
Length of first nucleic acid turn negative (days)	16.5 (21.5–11.5)	18.0 (19.0–16.0)	0.617
Treatment with antibiotics [cases (%)]	6 (37.5)	10 (34.5)	0.84
Use antiviral therapy [cases (%)]	7 (43.8)	24 (82.8)	0.016
Use of hormone therapy [cases (%)]	5 (31.3)	17 (58.6)	0.079
Treatment with neutralizing antibody [cases (%)]	9 (56.3)	17 (58.6)	0.878
Vaccinated with COVID-19 vaccine [cases (%)]	11 (68.8)	26 (89.7)	0.11

^a^Data are presented as median (IQR) or n (%). P-values are calculated by χ^2^-test or Mann-Whitney *U*-test between the re-positive group and the non-re-positive group.

Extreme value: In this table, one patient in the non-re-positive group was too young, only 33 days. In order to avoid errors, this patient was excluded from the statistics of the age of the non-re-positive group.

### Comparisons of T lymphocyte subsets in the peripheral blood between the re-positive and non-re-positive groups at admission

While the number of T lymphocyte subsets in the re-positive group was slightly higher than that in the non-re-positive group at admission, the difference was not statistically significant (*P* > 0.05; Table 2).

**Table 2:** Comparison of T lymphocyte subsets between the re-positive and non-re-positive groups^a^.

**Variables**	**Re-positive group (*n* = 16)**	**Non-re-positive group (*n* = 28)**	***P*-value**
Absolute number of lymphocytes/uL	1,191 (1,414–1,025)	952 (1,367–763)	0.311
Total absolute number of T lymphocytes/uL	761 (1,072–572)	706 (986–477)	0.348
Absolute number of suppressor/cytotoxic T lymphocytes/uL	322 (419–153)	250 (350–158)	0.414
Absolute number of helper/induced T lymphocytes/uL	403 (584–308)	381 (594–301)	0.836
**Nucleic acid CT value at admission (nasal swab)**			
ORF1ab gene	23.5 (30.0–19.3)	19.6 (25.1–18.3)	0.169
*N* gene	22.1 (33.4–16.1)	18.8 (25.4–15.5)	0.302

^a^Data are presented as median (IQR).

Missing Data: One patient in the non-re-positive group lacks data for the detection of T lymphocyte subsets.

### Comparisons of the nucleic acid CT values between the re-positive and non-re-positive groups

The median CT values for the viral ORF1ab and *N* genes were higher in the re-positive group than those in the non-re-positive group at admission, the difference was not statistically significant (*P* > 0.05; Table 2). The median nucleic acid CT values at the time of the first re-positive test in the re-positive group were significantly higher than they were at admission (*P* < 0.05; Table 3).

**Table 3:** Comparisons of nucleic acid CT values at admission and at the time of the first re-positive test in the re-positive group^a^.

**Group**	**ORF1ab gene ($\bar{x}$ ±s)**	***N* gene ($\bar{x}$ ±s)**
At the time of admission	23.5 (30.0–19.3)	22.1 (33.4–16.1)
At the time of re-positive	37.0 (39.7–35.2)	36.4 (39.3–35.2)
*z*-value	4.716	4.524
*P*-value	0.001	0.001

CT values, the cycle threshold value of SARS-CoV-2 RT-PCR.

^a^Data are presented as median (IQR).

### Comparisons of IgG and IgM antibody levels between the re-positive and non-re-positive groups

**Figure 2:**
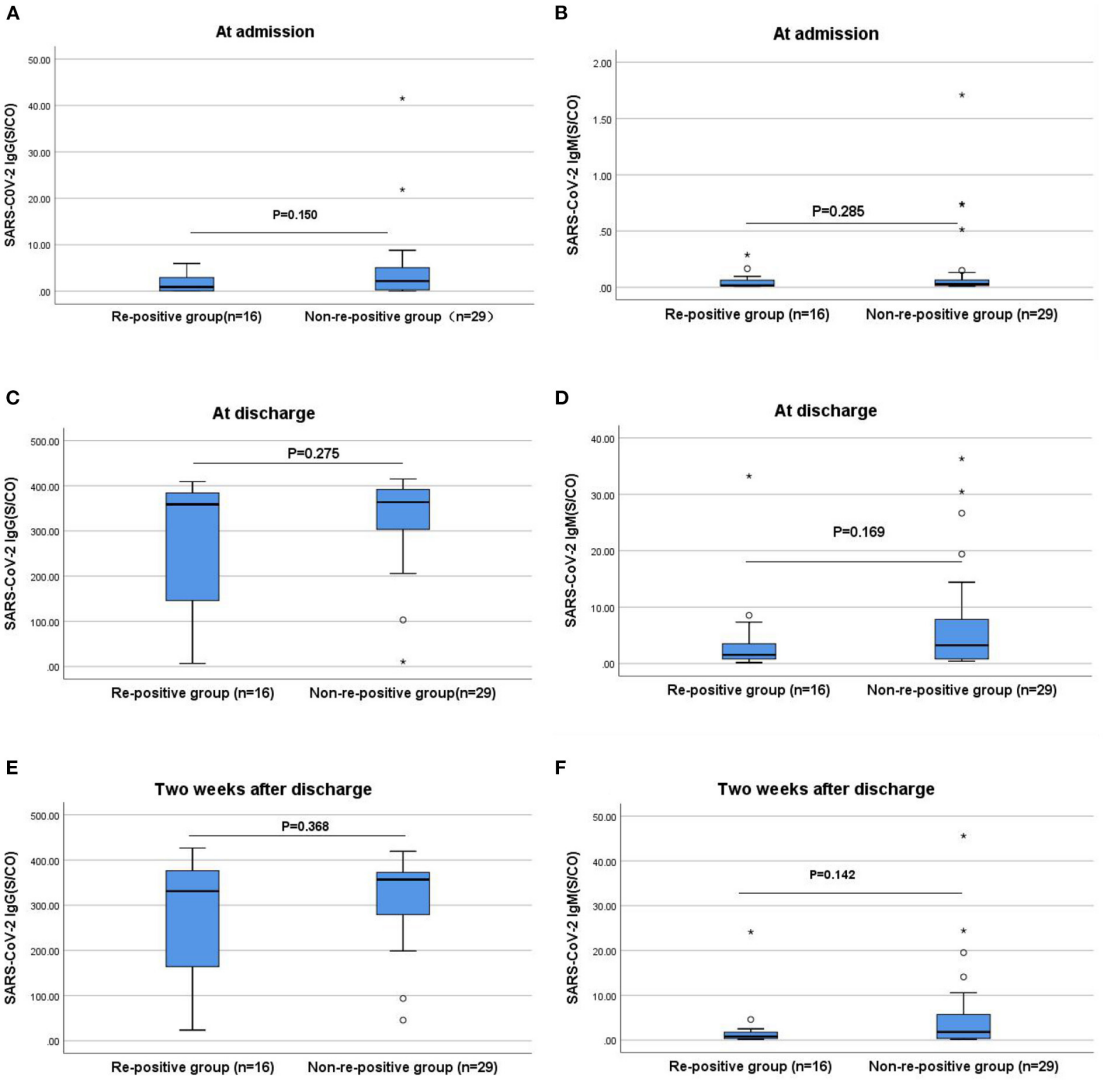
Comparisons of the differences in IgG and IgM antibody levels between the re-positive and non-re-positive groups at admission, discharge, and 2 weeks after discharge. **(A)** Comparison of IgG antibody levels between two groups at admission (*P* > 0.05). **(B)** Comparison of IgM antibody levels between two groups at admission (*P* > 0.05). **(C)** Comparison of IgG antibody levels between two groups at discharge (*P* > 0.05). **(D)** Comparison of IgM antibody levels between two groups at discharge (*P* > 0.05). **(E)** Comparison of IgG antibody levels between two groups two weeks after discharge (*P* > 0.05). **(F)** Comparison of IgM antibody levels between two groups two weeks after discharge (*P* > 0.05). The titers of IgG and IgM antibodies are analyzed by Mann-Whitney *U*-test and expressed by box chart. The symbol * indicates the outliers.

The antibody levels and antibody positivity rates at admission, discharge, and 2 weeks after discharge in the re-positive group were slightly lower than those in the non-re-positive group; however, the differences were not statistically significant (*P* > 0.05; Figure 2). At discharge, the median IgG antibody levels were significantly higher in patients treated with neutralization antibodies than those without. Of the 37 vaccinated patients, 23 cases who received neutralizing antibody therapy had significantly higher median IgG antibody levels than 14 cases who did not (IgG level: 383 vs. 282, *P* = 0.001). Among the eight patients who were not vaccinated, three patients who received neutralizing antibody treatment had significantly higher median IgG antibody levels than five patients who did not receive treatment (IgG level: 346 vs. 12.5, *P* = 0.036).

### Comparisons of clinical characteristics between the first re-positive time in the <7 and ≥7 days groups

The re-positive group was divided into two subgroups according to time to the first re-positive test (<7 and ≥7 days, *n* = 8 patients each). The average ages differed significantly between the <7 and ≥7 days groups (50 vs. 24, *P* < 0.05). Compared with the <7 days group, the ≥7 days group had a higher proportion of not using neutralizing antibody therapy (12.5% vs. 75%, *P* = 0.041).

### Comparisons of peripheral blood T lymphocyte subsets and nucleic acid CT between the <7 and ≥7 days groups

A comparison of T lymphocyte subsets at admission between the two groups showed a significantly higher absolute number of lymphocytes, total T lymphocytes, and auxiliary/induced T lymphocytes at admission in the ≥7 days group than in the <7 days group, and that the difference was statistically significant (*P* < 0.05). Moreover, further comparisons of the median lowest nucleic acid CT values during the re-positive period between the two groups showed that the median level of the ORF1ab gene in the ≥7 days group was lower than that in the <7 days group. The difference is not significant (*P* = 0.065; Table 4).

**Table 4:** Comparisons of laboratory data between the <7 and ≥7 days groups^a^.

**Variables**	** <7 d group (*n* = 8)**	**≥7d group (*n* = 8)**	***P*-value**
Absolute number of lymphocytes/uL	1,067 (1,276–456)	1,360 (1,807–1,160)	0.028
Total absolute number of T lymphocytes/uL	704 (832–328)	1,055 (1,331–736)	0.028
Absolute number of suppressor/cytotoxic T lymphocytes/uL	228 (423–98)	339 (419–216)	0.328
Absolute number of helper/induced T lymphocytes/uL	321 (472–201)	489 (777–395)	0.028
**Nucleic acid CT value at admission (throat swab)**			
ORF1ab gene	23.2 (28.8–21.2)	25.1 (27.2–21.8)	0.959
*N* gene	22.8 (29.4–18.9)	25.2 (28.6–19.6)	0.798
**Nucleic acid CT value at admission (nasal swab)**			
ORF1ab gene	22.4 (24.2–17.4)	26.8 (32.0–21.7)	0.195
*N* gene	21.9 (23.0–14.6)	27.4 (33.6–19.5)	0.442
**Nucleic acid CT value at the time of re-positive**			
ORF1ab gene	38.5 (39–35.9)	36.3 (37.9–32.8)	0.130
*N* gene	37.1 (39.7–36.1)	36.1 (38.8–32.3)	0.234
**Minimum nucleic acid CT value during re-positive period**			
ORF1ab gene	38.5 (39.8–35.9)	35.3 (37.9–31.6)	0.065
*N* gene	36.5 (39.7–35.1)	35.6 (38.7–31.8)	0.328

CT value, the cycle threshold value of SARS-CoV-2 RT-PCR.

^a^Data are presented as median (IQR). P-values are calculated by Mann-Whitney *U*-test.

Missing Data: One patient in the non-re-positive group lacks data for the detection of T lymphocyte subsets.

### Comparisons of IgG and IgM antibody levels between the <7 and ≥7 days groups

**Figure 3:**
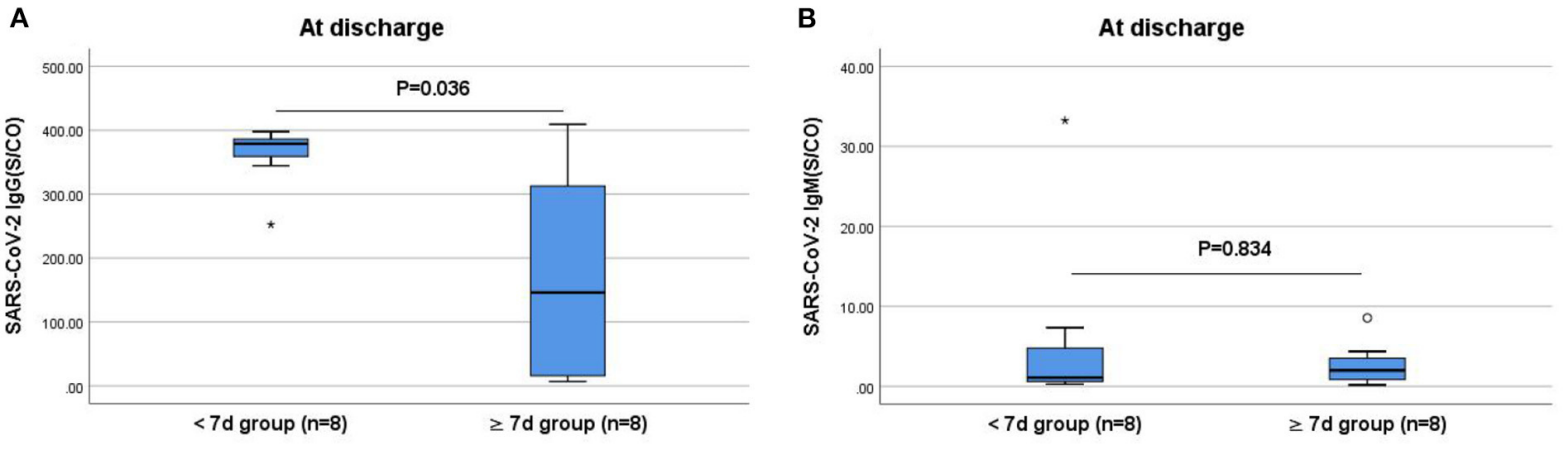
Comparisons of the difference in IgG and IgM antibody levels between the <7 and ≥7 days groups at discharge. **(A)** Comparison of IgG antibody levels at discharge between two groups (*P* < 0.05). **(B)** Comparison of IgM antibody levels at discharge between two groups (*P* > 0.05). The titers of IgG and IgM antibodies are analyzed by Mann-Whitney *U*-test and expressed by box chart. The symbol * indicates the outliers.

The IgG and IgM antibody levels when compared did not differ significantly between the ≥7 and <7 days groups at admission (*P* > 0.05). However, the IgG antibody levels at discharge were significantly lower in the ≥7 days group than those in the <7 days group (*P* < 0.05; Figure 3).

### Comparisons of clinical characteristics of the first re-positive time in the ≥7 days (*n* = 8) and non-re-positive (*n* = 29) groups

We compared the general and clinical data between the ≥7 days and non-re-positive groups. The age in the ≥7 days group was significantly lower than that in the non-re-positive group (16 vs. 53, *P* = 0.004). Four cases (50%) and three cases (10.3%) in the ≥7 days and non-re-positive groups, respectively had not received a COVID-19 vaccine.

### Comparisons of peripheral blood T lymphocyte subsets and nucleic acid CT values on admission between the ≥7 days and non-re-positive groups

The median absolute number of lymphocytes and total absolute number of T lymphocytes in the ≥7 days group was significantly higher than that in the non-re-positive group (*P* < 0.05). There were no significant differences in N gene levels of nasal swabs nucleic acid CT values between the two groups at admission; however, the ORF1ab gene levels in the ≥7 days group were significantly higher than those in the non-re-positive group (*P* < 0.05; Table 5).

**Table 5:** Comparisons of laboratory data between the ≥7 days and non-re-positive groups^a^.

**Variables**	**≥7 d group (*n* = 8)**	**Non re-positive group (*n* = 29)**	***P*-value**
Absolute number of lymphocytes/uL	1,360 (1,807–1,160)	952 (1,367–763)	0.027
Total absolute number of T lymphocytes/uL	1,055 (1,331–736)	706 (986–477)	0.04
Absolute number of suppressor/cytotoxic T lymphocytes/uL	339 (419–216)	249 (350–158)	0.116
Absolute number of helper/induced T lymphocytes/uL	489 (777–395)	381 (594–301)	0.145
**Nucleic acid CT value at admission (throat swab)**			
ORF1ab gene	25.1 (27.2–21.8)	23.7 (28.7–21.2)	0.957
*N* gene	25.2 (28.6–19.6)	23.5 (27.7–20.3)	0.871
**Nucleic acid CT value at admission (nasal swab)**			
ORF1ab gene	26.8 (32.0–21.7)	19.6 (25.1–18.3)	0.029
*N* gene	27.4 (33.6–19.5)	18.8 (25.4–15.5)	0.094

CT value, the cycle threshold value of SARS-CoV-2 RT-PCR.

^a^Data are presented as median (IQR). P-values are calculated by Mann-Whitney *U*-test.

Missing Data: one patient in the non-re-positive group lacks data for the detection of T lymphocyte subsets.

### Comparisons of IgG and IgM antibody levels between the ≥7 days and non-re-positive groups

**Figure 4:**
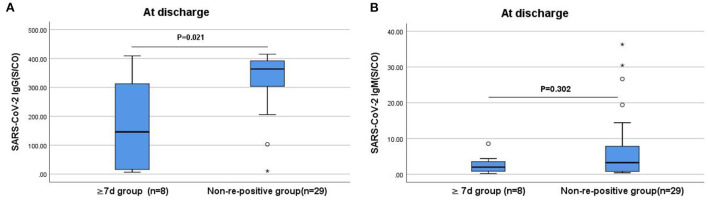
Comparison of the differences in IgG and IgM antibody levels between the ≥7 days and non-re-positive groups at discharge. **(A)** Comparison of IgG antibody levels at discharge between two groups (*P* < 0.05). **(B)** Comparison of IgM antibody levels at discharge between two groups (*P* > 0.05). The titers of IgG and IgM antibodies are analyzed by Mann-Whitney *U*-test and expressed by box chart. The symbol * indicates the outliers.

The IgG and IgM antibody levels at admission and the IgM antibody levels at discharge in the ≥7 days group were slightly lower than that in the non-re-positive group; however, the difference was not statistically significant. The IgG antibody levels in the ≥7 days group were significantly lower at discharge (*P* < 0.05; Figure 4).

## Discussion

The 75 patients diagnosed with COVID-19 infections by the original strain in the Ningxia Hui Autonomous Region between January 22 and March 16, 2020, were discharged from the hospital after 2 weeks and underwent a nucleic acid reexamination 4 weeks after discharge. Of these, six cases were re-positive, corresponding to a re-positivity rate of 8% (11). For the 45 patients infected with the Delta variant, nucleic acid re-positive began on the 3rd day after discharge, total 16 cases were re-positive, corresponding to a re-positivity rate of 35.6%. However, calculated in terms of 2 weeks and 2 weeks later, four cases were re-positive, corresponding to a positivity rate of 8.8%. Therefore, the re-positivity rate of the Delta variant strain was approximately the same as that of the original strain after 2 weeks. Unvaccinated children infected with the Delta variant were more likely to be re-positive. Among the five unvaccinated children, four cases were re-positive, corresponding to a re-positivity rate of 80%. Moreover, the age in the re-positive group was lower than that in the non-re-positive group, suggesting that the younger age group was more likely to show re-positivity (*P* < 0.05), which was consistent with the results of the study by An et al. (12). This may have been because a high proportion of children were in the re-positive group due to the low levels of protective antibodies in those who did not receive the COVID-19 vaccine. Their immature immune systems and lower levels of specific IgG, which may have failed to eliminate the virus from their body, thus making them prone to nucleic acid re-positivity during the recovery period. The situation has escalated due to the continuous variations of SARS-CoV-2, as well as the difficulty in epidemic prevention and control, hence greater protection of children is recommended. Wu et al. (13) suggested that residual virus may re-aggravate pathological changes in the lungs after antiviral drugs are discontinued and discharged from the hospital, leading to re-positivity. In the present study, a high proportion of patients in the re-positive group did not received antiviral therapy (*P* < 0.05). The antiviral therapy used in this group of patients was a combination of ribavirin and interferon, in accordance with the guidelines (8). Although no comparative studies have reported the efficacy of ribavirin and interferon therapy in SARS-CoV-2, we speculate that the antiviral therapy may have had a scavenging effect on the virus and that the patients who did not receive antiviral therapy or who used it only for a short period may not have been able to eliminate the virus from their bodies; therefore, the levels of the remaining virus may have increased after discharge, making these patients more prone to re-positivity.

The CT value of the nucleic acids, also known as the cycle threshold, reflects the amount of virus in the human body, with lower CT values indicating fewer cycles and a higher viral load in the body (14). A study of 137 patients infected with the original SARS-CoV-2 strain reported a median viral shedding period of 20–37 days (15). However, a study on the Delta variant found that PCR detection showed a lower cycle threshold and longer viral shedding period, indicating it's significantly enhanced transmission ability (16). In the present study, while the median nucleic acid CT values of the nasal and pharyngeal swabs on admission were higher in the re-positive group than those in the non-re-positive group, the difference was not statistically significant. However, the median nucleic acid CT value of the nasal swabs on admission in the ≥7 days group was significantly higher than that in the non-re-positive group (*P* < 0.05). Patients with a low viral load at admission may have had a longer intermittent detoxification period, resulting in a high nucleic acid re-positivity rate. The CT value of the nucleic acid in the re-positive group at the time of the first re-positive test (>35) was significantly higher than that at admission (*P* < 0.05). In addition, no symptoms or disease progression were observed in the re-positive cases; moreover, no cases of infections among the close contacts were reported, which suggested a lower viral load and a lower risk of transmission in re-positive patients. Yang et al. (17) also showed that patients in the recovery period were in the intermittent detoxification period and may have returned to a positive status rather than being re-infected. No live virus was isolated, and no viral genome fragments were detected in the re-positive patients, indicating a low risk of virus transmission from these patients. The present study (18, 19) demonstrated that the infectious virus produced was with very low titers when the CT value was ≥30, which indicated that others were less likely to be infected. A study (20) also showed that when the CT value is ≥ 30, the viral culture is mainly unsuccessful and the maximum CT value of virus-positive cultures is 34.3. Therefore, when nucleic acid CT value ≥ 35, basically no infectivity. However, it has been reported in a study, that a live virus was isolated from an immunodeficient patient with a minimum nucleic acid CT value of 15.6 during the re-positive period, suggesting that very few re-positive patients, especially immunocompromised individuals, may be at risk of infectivity (21). By comparing the clinical characteristics of the two subgroups, we found that the ≥7 days group showed a higher CT value of viral nucleic acid at the time of initial infection and lower IgG antibody level at discharge than the <7 days group. However, the median of the lowest viral nucleic acid CT value during the re-positive period was lower (35 vs. 38). Based on these results, we speculate that patients with re-positive after ≥7 days may be true re-positive. They reached the discharge standard (CT value ≥ 35) mentioned in Guidelines for Diagnosis and Treatment for Novel Coronavirus Pneumonia (Trial ninth edition) (22). A comprehensive evaluation of the infectivity of the re-positive patients in different re-positivity time periods was low, but in contrast, the CT value of nucleic acid in patients with later re-positivity time is lower and needs more attention. Meanwhile, in the ≥7 days group, two cases remained positive for >2 months; among these, one case became negative after 65 days, while the other case became negative after 74 days. One study (23) referred to these cases as long-term carriers and showed that they may exhibit long-term shedding of infectious SARS-CoV-2 and be at risk of transmission. However, this risk was shown to be lower than that of patients in the acute phase of infection. The results of our study support the notion that re-positive cases are caused by long-term but intermittent virus shedding. The intermittent detoxification time may be longer in patients with low virus loads at admission. Even if no live virus is present in convalescent patients, it takes some time to eliminate nucleic acid fragments. Some studies (24) have shown that the shedding of the infectious virus will drop to an undetectable level when the RNA load of the virus is low and neutralizing antibodies are present in the serum. Therefore, the results of the present study suggested indirectly determining the viral load in the body based on the change in nucleic acid CT value during the re-positive period to determine the necessity to isolate the virus and implement more strict management measures for the re-positive population with a high viral load or presence of live virus.

Of the 45 cases included in this study, 37 (82.2%) had received a COVID-19 vaccine, with children accounting for five (62.5%) of the eight unvaccinated cases. All types of vaccines were effective in reducing the incidence of COVID-19 associated severe illness and mortality (25). Patients with mild and common symptoms accounted for 97.8% of cases, which was attributed to the relatively high vaccination rate in the region. A previous study reported differences in the peripheral blood counts of CD3+, CD4+, and CD8+ T cells in patients with COVID-19, all of which decreased with increasing disease severity, and also that lymphocyte subset levels were helpful in assessing the disease and determining the prognosis (26). In the present study, while the T lymphocyte subsets in the re-positive group were higher than those in the non-re-positive group at admission, the difference was not statistically significant (*P* > 0.05). Further comparison showed that the median absolute number of lymphocytes and total absolute number of T lymphocytes in the ≥7 days group was significantly higher than that in the non-re-positive group (*P* < 0.05). In addition, the median nucleic acid CT values from the nasal and pharyngeal swabs on admission were higher in the re-positive group than those in the non-re-positive group (*P* > 0.05). The median nucleic acid CT value of the nasal swabs on admission in the ≥7 days group was also significantly higher than that in the non-re-positive group (*P* < 0.05). We speculated that the higher the nucleic acid CT value of Delta mutant, the lower the viral load, the weaker the inhibition of virus by the immune system, and the less obvious the decrease in T lymphocyte subset count. Although patients with a low viral load have less damage to the immune system, their detoxification may take longer. Since the T lymphocyte subsets were not detected in the peripheral blood of the re-positive group at the time of the first re-positive test, the determination of whether the number of T lymphocyte subsets on admission is related to the re-positive rate requires further study.

In this study, while the levels and positivity rates of the IgG and IgM antibodies in the re-positive group were lower than those in the non-re-positive group at admission, discharge, and 2 weeks after discharge, the differences were not statistically significant. Further comparison of the IgG antibody levels at discharge between the ≥7 and <7 days groups revealed that patients with lower IgG antibody levels at discharge took a longer time for re-positivity (*P* < 0.05). The IgG antibody level at discharge was significantly lower in the ≥7 days re-positive group compared with the non-re-positive group (*P* < 0.05), which may be attributed to the high proportion of unvaccinated patients, high nucleic acid CT values at admission, and low viral loads in the re-positive group. With low levels of protective antibodies in unvaccinated patients and weak immune responses at low viral loads, the levels of IgG and IgM antibodies are low. The specific IgG is a neutralizing antibody for which positivity and high titers suggest long-term protection after viral infection (27). Furthermore, stronger antibody responses to SARS-CoV-2 contribute to improved survival, and a protective effect that is independent of the disease severity. Maintaining high IgM and IgG antibody levels may be an indicator of rapid recovery (28). Our results showed that patients with lower IgG and IgM antibody levels at the time of discharge were more likely to be re-positive, especially patients with lower levels of IgG antibodies. Therefore, we recommend increased attention to changes in IgG and IgM antibody levels in patients with COVID-19 after discharge and in convalescent patients with low IgG antibody levels who are more likely to become re-positive.

Previous studies showed the weak effects of neutralizing antibody drugs on late-stage novel coronavirus infections and their important role mainly in the early treatment of COVID-19 (29). In the present study, the median IgG antibody levels of patients treated with neutralization antibody therapy were significantly higher than of those without the treatment at discharge and 2 weeks after discharge (*P* < 0.05), which suggested that neutralization antibody therapy might effectively improve IgG antibody levels in patients with COVID-19, especially those who are unvaccinated. In this study, the proportion of cases that did not receive neutralization antibody therapy in the ≥7 days group was higher than that in the <7 days group (75% vs. 12%, *P* = 0.041). The median IgG antibody level was also lower in the ≥7 days group than the <7 days group, which indirectly suggested that patients who took a later time to re-positivity had lower specific IgG levels *in vivo*, thus, a weaker ability to neutralize the virus. Therefore, viral replication led to nucleic acid re-positivity. And combined with the lower level of nucleic acid CT value at the time of re-positive. These may have been true re-positive cases; however, the correlation between neutralization antibody therapy and re-positivity requires further investigation.

### Limitations and strengths

The limitations of this study are as follows: First, this study only analyzed the clinical and epidemiological data of COVID-19 re-positive cases but could not obtain isolated virus samples; therefore, it was not possible to comprehensively evaluate the infectivity of the nucleic acid retest re-positive samples. Secondly, the sample size of this study was small and had limitations; therefore, the findings require further verification. Finally, the unified diagnostic criteria and management measures of COVID-19 re-positive patients should be further studied.

The strengths of this study include the dynamic analysis of IgG and IgM antibody levels, viral nucleic acid CT values at admission, and levels of T lymphocyte subsets to assess the risks of re-positivity among patients infected with the Delta variant infection, understand the re-positivity rate of Delta variant infection.

## Conclusion

In conclusion, our study discovered that: (1) The re-positivity rate of SARS-CoV-2 Delta mutant infection was higher, but the re-positivity rate remained constant after 2 weeks compared with the original strain. (2) Young people, patients who did not use antiviral therapy or had low IgG antibody levels at discharge were more likely to be re-positive. Additionally, the CT value of nucleic acid at the time of initial infection was higher in re-positive group. We speculated that the higher the CT value of nucleic acid at the time of initial infection, the longer the intermittent shedding time of the virus. (3) Re-positive patients were asymptomatic. The median CT value of nucleic acid was > 35 at the re-positive time, and the close contacts were not detected as positive. The overall transmission risk of re-positive patients is low. Due to this study lacking the isolation or culture of live viruses in re-positive patients, it is impossible to fully assess the infectivity of patients with re-positive, which can be further studied.

The following recommendations are made for the management of re-positive cases based on the findings of this study. To begin, hierarchical management of re-positive patients: (a) When there are no symptoms and signs and the CT value of the nucleic acid test is > 30 at the re-positive time, it is classified as low risk. Re-positive patients can be monitored at home. Keeping track of the symptoms and signs of re-positive patients, as well as their dynamic changes in nucleic acid CT value. However, patients and their families need regular nucleic acid reexamination. (b) When the CT value of nucleic acid test was ≤ 30, it is classified as high risk, and the risk of transmission was quickly assessed in combination with the course of the disease and the dynamic change of CT value. If there was a risk of transmission, it was managed according to the infected person, isolation measures and close person tracks were taken, and if necessary, live virus isolation or culture was done to clarify the risk of infection. Hierarchical management measures reduce the burden of managing re-positive cases and save medical resources. Secondly, there is uncertainty regarding the danger of transmission due to the incomplete elucidation of the re-positive mechanism in COVID-19 and the prevalence of SARS-CoV-2 variant strains. It can adopt multi-site joint sampling and enhance the number of nucleic acid tests conducted prior to discharge, both of which have practical guiding relevance for lowering the incidence of re-positives and the potential risk of community transmission. Finally, after the patients were re-positive, a study (30) revealed that the rate of sadness and sleeplessness considerably rose. The protection of re-positive patients' personal privacy and mental health advice should also be strengthened. In order to prevent panic, it is valuable to popularize the very low infectivity of COVID-19 re-positive patients to the masses.

## Data availability statement

The raw data supporting the conclusions of this article will be made available by the authors, without undue reservation.

## Ethics statement

The studies involving human participants were reviewed and approved by General Hospital of Ningxia Medical University (KYLL-2021-968). The patients/participants provided their written informed consent to participate in this study. Written informed consent was obtained from the individual(s) for the publication of any potentially identifiable images or data included in this article.

## Author contributions

WZ: conceptualization, methodology, and validation. JW and S-XZ: data curation and writing original draft preparation. J-RN, L-LZ, Y-HZ, J-JC, LG, MY, and Y-TL: writing review and editing. All authors contributed to the article and approved the submitted version.
