# Supplementary material for: A comparative study of virus nucleic acid re-positive and non-re-positive patients infected with SARS-CoV-2 Delta variant strain in the Ningxia Hui Autonomous Region
Source: Front Public Health. 2022 Dec 13;10:1023797. doi: 10.3389/fpubh.2022.1023797 (PMC9792667; doi:10.3389/fpubh.2022.1023797)

**Supplementary Materials**

The small sample size of this study required supplementation of the sample size test. This study used the single sample rate comparison (single group target value method) to calculate the sample size. The results of studies^[5,6]^ on re-positivity of the original COVID-19 strain at home and abroad showed re-positivity rates of approximately 19–21%; therefore, the present study assumed a re-positivity rate of 20.0% (p_1_). With reference to the latest Chinese literature^[7]^ on the re-positivity of Delta variant infection, the re-positivity rate of the Delta variant infection was about 61.4%%. So the baseline rate was set at 61.4%(p_0_). The sample size was calculated based on an α of 0.01 and a degree of assurance 1-β controlled at 90%.

Given a significance level α=0.01, β=0.1, test power 1-β=0.9, p_0_=61.4%, p_1_=20.0%, the following formula was used:

n=*
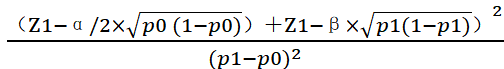
*

*The calculated sample size was 19 cases. Considering the lack of sample size, the sample size was increased by 20%; thus, the required sample size was at least 24 cases. At the same time, we also used PASS 15.0.5 software to verify, which was consistent with the sample size calculated by our formula. The sample size in the present study was 45 cases; which provided statistical significance.”*


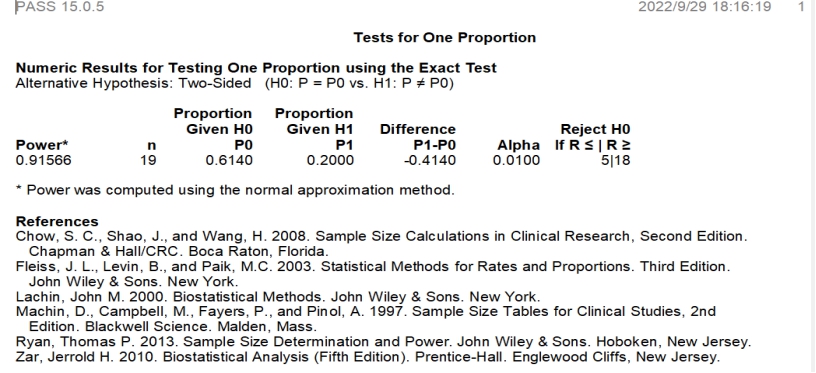

Supplement: Supplementary file 1 [file Data_Sheet_1.docx]
